# Abundant Lipid and Protein Components of Drusen

**DOI:** 10.1371/journal.pone.0010329

**Published:** 2010-04-23

**Authors:** Lan Wang, Mark E. Clark, David K. Crossman, Kyoko Kojima, Jeffrey D. Messinger, James A. Mobley, Christine A. Curcio

**Affiliations:** 1 Department of Ophthalmology, University of Alabama at Birmingham, Birmingham, Alabama, United States of America; 2 Department of Microbiology, University of Alabama at Birmingham, Birmingham, Alabama, United States of America; 3 Department of Epidemiology, University of Alabama at Birmingham, Birmingham, Alabama, United States of America; 4 Department of Surgery, University of Alabama at Birmingham, Birmingham, Alabama, United States of America; University of Oldenburg, Germany

## Abstract

**Background:**

Drusen are extracellular lesions characteristic of aging and age-related maculopathy, a major retinal disease of the elderly. We determined the relative proportions of lipids and proteins in drusen capped with retinal pigment epithelium (RPE) and in RPE isolated from non-macular regions of 36 human retinas with grossly normal maculas obtained <6 hr after death.

**Methodology/Principal Findings:**

Druse pellets were examined by light and electron microscopy. Component proteins were extracted using novel methods for preserved tissues, separated, subjected to tryptic digestion and LC-MS(MS)^2^ analysis using an ion trap mass spectrometer, and identified with reference to databases. Lipid classes were separated using thin layer chromatography and quantified by densitometry. Major druse components were esterified cholesterol (EC), phosphatidylcholine (PC), and protein (37.5±13.7, 36.9±12.9, and 43.0±11.5 ng/druse, respectively). Lipid-containing particles (median diameter, 77 nm) occupied 37–44% of druse volume. Major proteins include vitronectin, complement component 9, apoE, and clusterin, previously seen in drusen, and ATP synthase subunit β, scavenger receptor B2, and retinol dehydrogenase 5, previously seen in RPE. Drusen and RPE had similar protein profiles, with higher intensities and greater variability in drusen. C8, part of the complement membrane attack complex, was localized in drusen by immunofluorescence.

**Conclusions/Significance:**

At least 40% of druse content is comprised by lipids dominated by EC and PC, 2 components that are potentially accounted for by just one pathway, the secretion of lipoproteins by RPE. Manipulating genes encoding apolipoprotein pathways would be a fruitful approach to producing drusen with high EC content in laboratory animals. Therapies that directly mitigate drusen should prepare for the substantial volume of neutral lipids. The catalog of major druse proteins is nearing completion.

## Introduction

Age-related maculopathy (ARM), a retinal disease affecting ∼10 million older Americans, involves 4 distinct layers of the outer retina: photoreceptors, retinal pigment epithelium (RPE, a layer of nurse cells dedicated to photoreceptor health), Bruch's membrane (BrM, a 5-layer vessel wall underlying the RPE), and choriocapillaris (capillaries in the vascular bed with the body's highest blood flow). Drusen are focal deposits of extracellular debris located between the basal lamina of the RPE and the inner collagenous layer of BrM [1], [2]. Found with advanced age in normal human eyes [3], [4], they are regarded as hallmarks of the underlying degeneration of ARM. Numerous small hard drusen increase significantly the incidence of large soft drusen and RPE abnormalities that are, in turn, more likely to progress to advanced disease [5]. Discrete and isolatable lesions amenable to multiple assays, drusen are known to contain carbohydrates, zinc, and at least 129 different proteins, including apolipoproteins and excluding extracellular matrix [6], [7], [8], [9], [10], [11], [12]. Druse proteins involved with inflammation and innate immunity (e.g., amyloid-β, immunoglobulin light chains, factor X, C3, C5b-9 complex) have received particular attention because sequence variants in several complement proteins are associated with increased ARM risk [13], [14], [15], [16].

Virtually all drusen contain histochemically detectable lipid [17], [18], [19], [20], [21], including cholesterol in 2 chemical forms, unesterified (UC) and esterified to a long chain fatty acid (esterified cholesterol, EC) [22]. EC and UC also accumulate in normal human BrM throughout adulthood [19], [20], [23], of note because aging is the largest risk factor for ARM. Elsewhere [24], [25] we summarized multiple lines of laboratory and clinical evidence supporting the hypothesis that this accumulation can be attributed to the retention of lipoprotein particles, 60–80 nm in diameter and containing abundant EC, UC, phosphatidylcholine (PC), and apolipoprotein B. The RPE is believed to secrete the lipoprotein accumulating in BrM, without excluding the possibility that lipoproteins of hepatic or intestinal origin arriving by plasma also contribute. BrM lipoproteins are rich in the fatty acid linoleate and poor in docosahexaenoate, and their density fraction contains apolipoproteins B, A-I, and E. Ultrastructural studies indicate that the concentration of lipoproteins is highest in the same tissue compartment (inner BrM) as drusen and basal linear deposit, a diffusely distributed drusenoid material specific to ARM [26]. ARM lesion formation has thus been conceptualized as sharing mechanisms with atherosclerotic plaque formation, with a key difference being that lipoproteins retained in BrM are of intra-ocular origin unlike the plasma LDL retained in large arteries [24], [25].

Although drusen are often described as lipid-rich or lipid containing, the exact proportion of protein and lipid in drusen is unknown. The actual value of this proportion is important for understanding how ARM-specific lesions form. Knowledge about component abundance could direct attention to major contributing pathways in order to build better model drusen in laboratory animals or to improve the molecular basis for mitigating or eliminating drusen in patients. In this study we isolated drusen with overlying caps of RPE, quantified total protein, identified individual proteins by mass spectrometry, and quantified neutral and polar lipid classes by thin layer chromatography. We analyzed drusen from extra-macular (peripheral) retina from aged normal eyes, as we have done previously [27], [28], [29], in order to maximize sample size for our assays. Extra-macular retina represents 90% of total retinal area and thus contains the numerical majority of drusen, even in ARM eyes [30], [31]. We elected to defer study of a distinct druse sub-type in ARM macula associated with risk for disease progression (“soft”) [5], [32] for future studies with more sensitive assays. Here we calculated mass of major components on a per druse basis, unlike others who estimated druse component abundance by the number of affected eyes (e.g., [33]) or the number of affected drusen within an eye [10]. We find that the lipids EC and PC may together account at least 40% of druse volume.

## Methods

### Donor eyes and isolation of drusen

Institutional Review at the University of Alabama at Birmingham approved our use of human tissues. Eyes were obtained from eye bank donors ≤6 hrs of death. RPE/choroid eyecups used for total protein measurements were snap-frozen within scleral shells using liquid nitrogen and stored at −80°C. Eyes used for all other assays were preserved by immersion in either 4% paraformaldehyde or 1% paraformaldehyde/2.5% glutaraldehyde (**Table 1**), both in 0.1 M phosphate buffer (PB), for 24 hrs following corneal excision and stored in 1% paraformaldehyde at 4°C until used. Eyes had grossly normal maculas and were chosen for use on the basis of druse abundance in the peripheral retina. Most came from older donors. Eyes chosen for use for protein identification by mass spectrometry included several 7^th^ decade donors and therefore had a younger mean age than the other groups [34].

**Table 1:** Summary of Eyes Used.

Purpose	#Eyes	Age(Mean ± SD)	Gender(F/M)	# RPE-cappedDrusen(Mean ± SD)	Preservative	Table code
TEM	2	79.5±2.1	2/0	235±108	Fix 2	
NL (TLC)	14	77.1±10.6	5/9	263±78	Fix 2	Table 2, 1–7*
PL (TLC)	7	78.6±9.7	6/1	310±147	Fix 2	Table 2, 8–14
Protein (BCA)	7	88.0±6.5	4/2	386±92	Frozen	Table 4, 15–21
Protein (MS)**	6	66.2±14.7	3/3	152±70	Fix 1	

Abbreviations: TEM: transmission electronic microscopy; BCA: bicinchoninic acid; MS: mass spectrometry; TLC: thin layer chromatography; NL: neutral lipid; PL: polar lipid; Fix 1: 4% paraformaldehyde in 0.1M phosphate buffer; Fix 2: 2.5% glutaraldehyde and 1% paraformaldehyde in 0.1 M phosphate buffer.

* 14 eyes were assayed in 2 groups, with similar results; one group is reported.

** 6 for RPE-capped drusen, 5 for RPE.

All RPE-capped drusen were extra-macular and were collected with a clearance of 3 mm from the outer circumference of the 6 mm-diameter macula. Drusen were mobilized from BrM with a borosilicate pipette under stereomicroscopic guidance, herded into groups, drawn into the pipette, placed into 1 ml vials containing PB, and gently spun at 10,000 rpm to create a pellet. All drusen within a peripheral retinal quadrant (1–2 quadrants per eye) were harvested, without regard to size or type. Our target for the number of drusen per eye was guided by previous experience with enzymatic cholesterol assays [28]. Drusen were counted, as they were isolated. Co-author LW collected drusen used for total protein determinations, and co-author MEC isolated all others. The volume occupied by drusen and RPE in pellets of RPE-capped drusen was estimated from the area fraction of these components in tissue cryosections [27], [28], [35]. We determined cross-sectional areas of RPE and drusen from differential interference contrast images, using a digitizing tablet and IP Lab (Wacom Technologies; BioVision Technologies, Exton PA). To allow comparison of a mixed sample (RPE-capped drusen) with RPE alone, RPE in druse-free areas were also collected from the same eyes.

### Transmission electron microscopy

To facilitate handling, PB-washed drusen were covered with a 0.75%-agarose/5%-sucrose solution and refrigerated to form solid agarose-encased pellets. Pellets were either extracted for 1 hr with chloroform/methanol (2∶1) [36] or left untreated prior to further processing. Pellets were trimmed, post-fixed in 1% osmium in 0.1 M sodium cacodylate buffer, 1% tannic acid, and 1% paraphenylenediamine (OTAP method [22], [37]), dehydrated through ethanol and propylene oxide, and embedded in epoxy resin (PolyBed 812; Polysciences, Warrington PA). One-µm-thick sections stained with 1% toluidine-O-blue were imaged with a light microscope (Eclipse 80i; Nikon, Melville NY), 60× oil-immersion objective, and digital camera (Retiga 4000R Fast; Q Imaging, Burnaby, BC, Canada) [29]. Silver gold sections were viewed on a JEOL1200 EXII electron microscope (JEOL USA, Peabody MA) and imaged with an AMT-XR 40 camera (Advanced Microscopy Techniques, Danvers MA).

Two sets of measurements were made using digital electron micrographs (20,000× original magnification, 2550×3300 pixel tiff format files). First, the proportion of electron-dense lipid-containing material in individual drusen was calculated using the segmentation functions in IP Lab. From the Analyze\Segmentation menu, the lower bound of the intensity threshold was set at 1 to include small electron-dense particles, and the upper bound was set empirically to include all large particles. The Modify Segment\Erode function was used to separate confluent particles, and then the total area of particles was determined and expressed as percentage of total druse area. Second, the diameter of ∼900 individual electron-dense particles was determined by tracing with the digitizing tablet and calculating equivalent diameter according to the formula for a circle.

### Thin layer chromatography (TLC), one-dimensional, and densitometry for lipids

Lipids were extracted from RPE-capped drusen and RPE using chloroform-methanol (2∶1 v/v) [36] as described [38], [39]. Aliquots of organic phase were evaporated under nitrogen, re-solubilized in chloroform, and applied to TLC plates (LHPKD silica gel 60A, Whatman). To separate neutral lipids EC, triglyceride (TG), UC, and fatty acids (FA), plates were developed in petroleum ether: diethyl ether: acetic acid (84∶15∶1). To separate polar lipids PC and sphingomyelin (SPM), plates were developed in chloroform: methanol: ammonium hydroxide (65∶25∶4). Plates were sprayed with 3% copper acetate in 8% phosphoric acid solution and heated to reveal bands. Standards were chloroform-solubilized 1,2-dioleoyl-sn-glycero-3-phosphocholine, oleate, triolein, cholesteryl oleate, SPM esterified to mostly palmitate (16∶0), and UC (SPM from Avanti Polar Lipids, Alabaster AL; others from Sigma). Each plate containing samples contained standards run at five dilutions (1, 1∶2, 1∶4, 1∶8, 1∶16) in order to generate a standard line. Plates were scanned, bands of samples and standards defined, and densities measured using an ImageQuant 400 Scan CCD imaging system and ImageQuant 400 Capture software (version 1.0.0, GE Healthcare, Piscataway NJ). Densities were converted to concentrations on a per plate basis using the standard line for that plate and Excel (Microsoft). In the tables, we report “total measured lipids,” because certain lipid classes, e.g., phosphatidylethanolamine, were not assayed.

### Total protein in RPE-capped drusen

Proteins were extracted from fresh-frozen RPE-capped drusen by T-PER® Tissue Protein Extraction Reagent (catalog # 78510, Pierce Inc, Rockford IL). Protein concentration was measured using bicinchoninic acid protein assay kits (catalog #23227, Pierce Inc) according to the manufacturer's instructions. Briefly, 100 µl of protein extraction reagent were added into RPE-capped drusen samples and homogenized. Samples were centrifuged at 10,000×g for 5 minutes to pellet tissue debris, and supernatant was collected. Duplicate samples at 1∶5 and 1∶10 dilutions were measured using a microplate reader (Model V Max; Molecular Devices, now MDS Analytical Technologies). We report the average of these 2 replicates, which were highly similar.

### Identification of proteins via mass spectrometry and bioinformatics

#### Drusen Protein Extraction

Following harvesting, druse samples were kept in PB saline (PBS) at 4°C for <3 wk. All steps occurred at room temperature unless noted. Each sample was washed three times in PBS and centrifuged at 14,000×g for 2 min, and the PBS discarded. Proteins were extracted using the Qproteome FFPE Tissue kit (Qiagen) following the manufacturer's instructions, with modifications necessitated by the use of paraformaldehyde-fixed tissues. Each sample was re-suspended in 25 µL of Qiagen EXB, incubated at 100°C for 20 min and then at 80°C, 750 rpm for 2 hr. The samples were centrifuged at 4°C, 14,000×g for 15 min. Supernatant containing extracted proteins was transferred to a fresh tube. Protein content was quantified using EZQuant (Invitrogen). Two hundred ng of protein per sample was separated on a 4–12% Novex Tris-Glycine gel (Invitrogen) at a constant 125V for 15 min. The gel was stained overnight with Colloidal Blue (Invitrogen) and de-stained in distilled water for 3 hr. One intense band per lane was excised and digested overnight with trypsin (Promega) following the manufacturer's instructions. Digests were extracted using 15 µL of 60% acetonitrile/0.1% trifluoroacetic acid. Extracts were dried with a speed vacuum and reconstituted in 10 µL of 5% acetonitrile/0.1% formic acid. The entire extract of each sample was used for mass spectrometry, as described below.

#### Protein Identification

Extracted and de-crosslinked proteins were subjected to standard analytic techniques. LC-MS(MS)^2^ analysis of the tryptic digest peptides was performed using a ThermoFinnigan LTQ-XL ion trap mass spectrometer equipped with a Thermo MicroAS autosampler and Thermo Surveyor HPLC pump, Nanospray source, and Xcalibur 1.4 instrument control (ThermoFinnigan, San Jose, CA). Peptide fractions were diluted by a factor of 10 in 0.1% formic acid prior to separation on a packed capillary tip, 100 µ×11 cm, with C18 resin (Jupiter C18, 5 µ, 300 Angstroms, Phenomenex, Torrance, CA). The flow rate during the solid phase extraction phase of the gradient was 3 µL/min and 500 nL/min during the separation phase. Mobile phase A was 0.1% formic acid, mobile phase B was acetonitrile with 0.1% formic acid. A 95 min gradient was performed with a 15 min washing period (100% A for the first 10 min followed by a gradient to 98% A at 15 min) to allow for solid phase extraction and removal of any residual salts. After the initial washing period, a 60 min gradient was performed where the first 35 min was a slow, linear gradient from 98% A to 75% A, followed by a faster gradient to 10% A at 65 min and an isocratic phase at 10% A to 75 min. MS/MS scans were acquired using an isolation width of 2 amu, an activation time of 30 ms, and activation Q of 0.250 and 30% normalized collision energy using 1 micro-scan and maximum injection time of 100 ms for each scan. The mass spectrometer was tuned prior to analysis using the synthetic peptide TpepK (AVAGKAGAR). Typical tune parameters were spray voltage = 1.8 kV, capillary temperature = 150°C, capillary voltage = 50 V, and tube lens = 100 V. The MS/MS spectra of the peptides were acquired using data-dependent scanning in which one full MS spectrum using a mass range of 400–2000 amu was followed by three MS/MS spectra.

#### Database searches, statistical analysis, and systems biology

Proteins were searched in species-specific subsets of the UniRef database. Tandem mass spectrometry data were converted to mzXML format using instrument-specific conversion software (Institute for Systems Biology, Seattle WA; Fred Hutchinson Cancer Center) and run separately through SEQUEST (ThermoFisher), X!TANDEM (Global Proteome Machine Organization), and MASCOT (Matrix Science Inc., Boston MA) software. Top-matching algorithms from all 3 packages were utilized in order to increase confidence in protein identifications and decrease the propensity for false negatives. Combined data were analyzed using Protein Prophet (Institute for Systems Biology) to determine a best fit and confidence level for a specific peptide fragmentation pattern.

Further analysis used Refiner MS and Analyst software (Expressionist Genedata) to align mass and time tags of ion plots generated from the post LCMS run, followed by common statistical analysis using Analyst and manual input of threshold values. Selection of important proteins utilized common non-parametric statistical tools (Kruskal-Wallis, Fisher's exact test, and permutation t-test). Proteins were considered important based on significance values obtained with these tests, fold change, and ability to identify the same peptide with high confidence in 50% or greater in either RPE-capped drusen or RPE. Spectral count intensities from mass spectrometry for each of 20 proteins from RPE-capped drusen (n = 6 eyes) and RPE (5/6 eyes) were exported to an Excel spreadsheet. Ion intensity data were also imported into the program Mayday (version 2.9, Tübingen, Germany), log transformed (base 10), and visualized using the enhanced heat map feature of this program. A list of UniRef referenced IDs for the most important proteins were uploaded to Ingenuity Pathways Analysis (www.ingenuity.com), which maps all proteins to previously referenced cellular components indexed in the Gene Ontology databases (www.geneontology.org).

### Indirect immunofluorescence

Cryosections of isolated drusen were used for immunofluorescence performed as described [27]. Goat antiserum to human C8 (Quidel, cat# A309) was used at a concentration of 1∶200. Identical concentrations of goat IgG were used on negative control sections. Slides were incubated with biotinylated anti-goat IgG 1∶500 for 2 hr at room temperature, washed, and incubated with rhodamine Red-X-conjugated streptavidin (1∶500; Jackson ImmunoResearch, West Grove PA). After washing, coverslips were mounted with Aqua Poly/Mount (Polysciences, Inc., cat# 526956. Sections were examined with 10× planapo and 40× plan fluor objectives and filter cubes for rhodamine and autofluorescence (in nanometers, excitation-dichroic-barrier, 540/25–565-630/60 and 480/30 –505-535/40, respectively).

### Determining lipid and protein content of RPE-capped drusen

To compare protein and lipid components of RPE-capped drusen, results of bicinchoninic acid and TLC assays were converted to units of ng per druse. Protein concentrations were originally expressed in terms of mg albumin equivalents per ml of extract from a known number of drusen. The concentration of different lipid classes detected by TLC, originally expressed in nmole/µL [40] was converted to ng/ml using the molecular weight of standard lipids for each class (i.e., cholesteryl oleate for EC), and then divided by the number of drusen in the sample. To compare compositional data from drusen, which are countable, and RPE, which is not, measures of content were normalized by dry weight.

## Results

A total of 36 eyes from 36 adult donors with grossly normal maculas were used for different assays (**Table 1**). The number of RPE-capped drusen harvested per eye ranged from 152 to 386.

**Figure 1:**
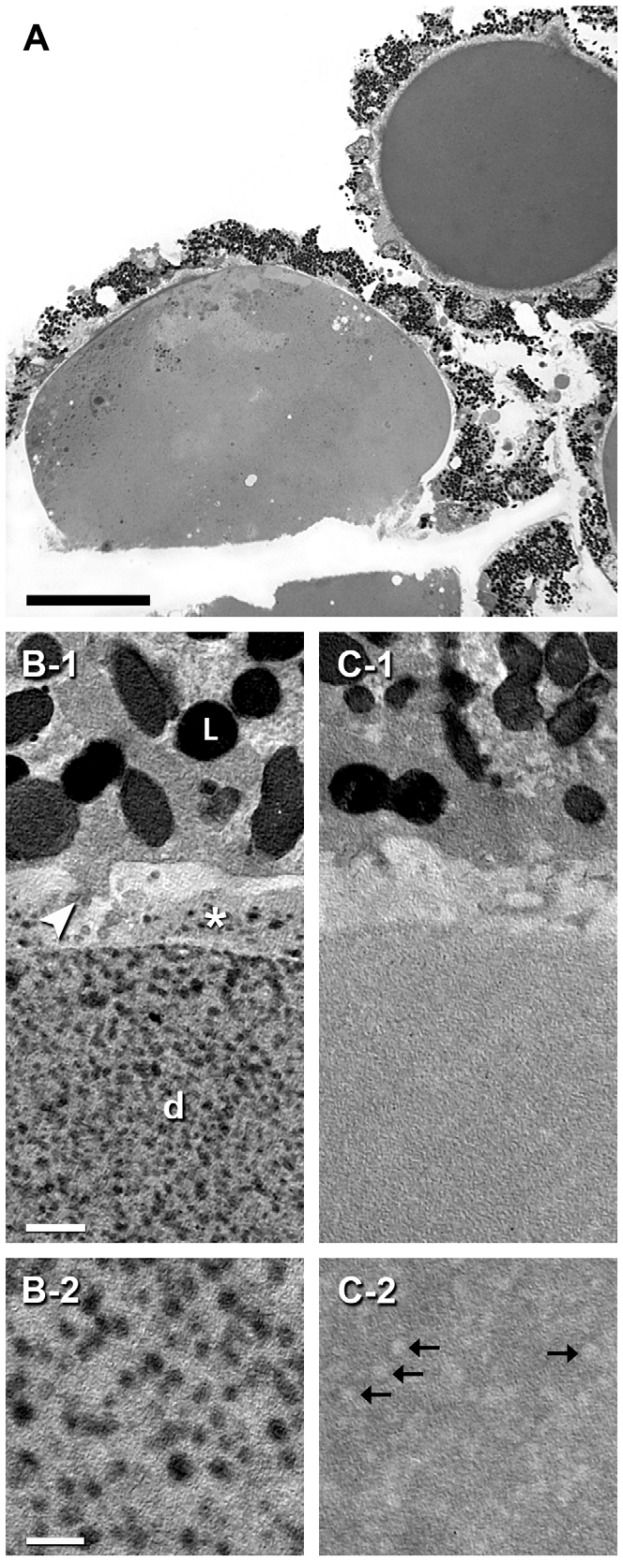
Lipid localization in isolated RPE-capped drusen. **A.** Light micrograph of RPE-capped drusen, isolated from extra-macular retina, pelleted, post-fixed by the OTAP method, and sectioned (1 µm). Two drusen in the panel are both considered hard. Bar, 50 µm. **B, C.** Transmission electron micrographs of RPE-capped drusen that are either untreated (B-1, B-2) or extracted with chloroform-methanol to remove lipids (C-1, C-2). RPE is at the top of B-1 and C-1. **B-1, B-2.** Drusen have abundant electron-dense (dark) lipid droplets. L, lipofuscin granule; d, druse interior; arrowhead, basal infolding; asterisk, basal laminar deposit. Bar in B-1, 1 µm. Bar in B-2, 200 nm. **C-1, C-2.** Lipid droplets are removed by chloroform-methanol extraction, leaving electron-lucent profiles (C-2).

**Figure 2:**
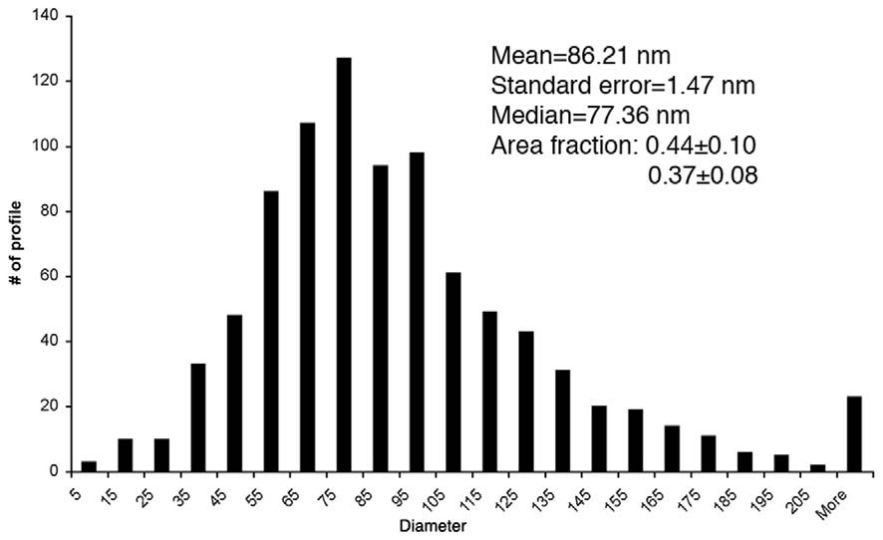
Lipid-containing particles in drusen. Electron-dense profiles were measured by digital planimetry in electron micrographs of drusen like Figure 1B-1, B-2, and equivalent diameters were determined. Descriptive statistics for this population of ∼900 particles are shown. Area fraction, the proportion of druse cross-sectional area occupied by electron-dense lipid, is reported for 2 eyes. See Methods for measurement details.

The morphology of pelleted RPE-capped drusen is illustrated in **Figure 1**. Drusen are randomly oriented in these sections. Most drusen are of the hard type, i.e., they are dome shaped with solid interiors and homogeneous contents, and a median diameter of 47 µm [29]. The RPE layer is intact overlying the druse, and because outer segments are occasionally attached, we infer that the entire apical to basal extent of RPE is present. By measuring the cross-sectional areas of drusen and RPE in sections of pelleted RPE-capped drusen (see Methods), we determined that the component volume fraction was 62.4%±12.8% for drusen and 37.6%±12.81 for RPE. Thus, assays of RPE-capped drusen described below are dominated by values for druse contents. In electron micrographs of OTAP prepared drusen, lipid-containing components take the form of spherical or ovoid particles that are electron dense and dispersed (**Figure 1B-1, B-2**). That these components are lipid containing is demonstrated by treatment with the lipid solvent chloroform – methanol, which converted areas of electron-density to electron-lucent spaces (**Figure 1C-1, C-2**). The lipid-containing components were quantified individually and in the aggregate in **Figure 2**. Particle diameters varied across a single-mode, positively skewed distribution with a median of 77.4 nm, with some profiles >200 nm. The area fraction of the electron-dense lipid containing material within individual drusen was 0.44 and 0.37 in two eyes.

To determine the lipid components of RPE-capped drusen, lesions were extracted with chloroform – methanol, and these extracts were separated using solvent systems for neutral lipids and polar lipids. **Table 2** shows that mean total lipid measured per RPE-capped druse was 112.42 ng/druse. While cognizant that combining data from these two assays has limitations, we can draw some conclusions. The polar lipids (PC and SPM, mean 61.13 ng/druse) were more abundant than the neutral lipids (EC, TG, FA, and UC, mean 51.30 ng/druse) in 4/7 eyes and less abundant in 3/7 eyes, indicating no strong trend towards dominance by one group. The two most abundant lipids measurable by these assays were EC and PC (37.47 and 36.89 ng/druse, respectively). Within the neutral lipids, EC was the dominant class, with values 4-fold or higher than the next highest class (FA). Within the polar lipids, PC was more abundant than SPM by 50% (paired t-test, p = 0.008).

**Table 2:** Measured Lipid Components in RPE-capped Drusen (ng/druse).

Case #	EC	TG	FA	UC	Total MeasuredNeutral Lipids	Case #	SPM	PC	Total MeasuredPolar Lipids	TotalMeasured Lipids
**1**	30.76	0.00	8.13	3.93	42.82	**8**	19.06	47.05	66.11	108.93
**2**	65.59	0.00	14.56	7.17	87.32	**9**	25.46	31.14	56.60	143.92
**3**	26.88	2.59	5.85	2.80	38.12	**10**	19.89	28.89	48.78	86.88
**4**	26.35	4.45	7.71	3.89	42.40	**11**	30.43	54.67	85.10	127.50
**5**	41.68	0.00	8.41	3.68	53.77	**12**	23.31	27.54	50.85	104.61
**6**	31.71	0.00	7.44	3.65	42.80	**13**	18.31	20.72	39.03	81.83
**7**	39.29	0.00	8.51	4.05	51.85	**14**	33.23	48.19	81.42	133.27
Mean	37.47	1.01	8.66	4.17	51.30	Mean	24.24	36.89	61.13	112.42
SD	13.70	1.80	2.75	1.39	16.84	SD	5.81	12.86	17.22	23.51

**Figure 3:**
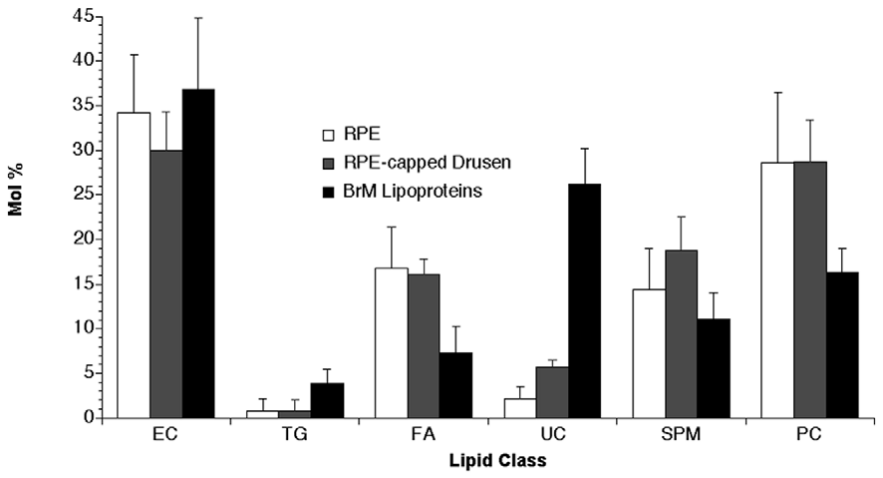
Lipid Composition. Mole% of major lipid classes in RPE-capped drusen, RPE, as determined by thin-layer chromatography and densitometry compared to that in BrM lipoprotein particles [39]. Abbreviations for 6 lipid classes are given in the notes to Table 2. As described in the Methods, EC, TG, FA, and UC were separated by a petroleum ether: diethyl ether: acetic acid solvent system, and SPM and PC were separated by chloroform: methanol: ammonium hydroxide.

To compare lipid profiles of RPE-capped drusen and RPE from the same eyes, lipid classes detected by TLC were expressed as percentages of dry weight, pooled across all eyes, in **Table 3**. While the same classes were detected in both samples, they differed distinctly, as 4.82% of the dry weight of RPE-capped drusen was EC, compared to 1.18% for RPE. For both neutral and polar lipids, RPE-capped drusen were enriched 4–6 fold relative to RPE. To compare lipid profiles of RPE-capped drusen, RPE, and previously published values for isolated BrM particles assayed by preparative liquid chromatography/gas chromatography [39], the mole percent of each class relative to the total of the 6 lipid classes measured is shown in **Figure 3**. All three samples were highly enriched in EC (30–35%). RPE and RPE-capped drusen, measured in this study, resembled each other but differed from BrM particles by being more enriched in FA and PC (by factors of ∼2), and much less enriched in UC.

**Table 3:** Lipid Fractions of RPE-capped Drusen and RPE (% of dry weight).

		RPE-cappedDrusen	RPE	Ratio(RPE-capped drusen/RPE)
Neutral Lipid	EC	4.82%	1.18%	4.08
	TG	0.19%	0.04%	4.75
	FA	1.59%	0.29%	5.48
	UC	0.52%	0.10%	5.20
	Total Measured NL	7.11%	1.60%	4.44
Polar Lipid	SPM	3.72%	0.57%	6.53
	PC	5.38%	0.92%	5.85
	Total Measured PL	9.10%	1.49%	6.11

**Figure 4:**
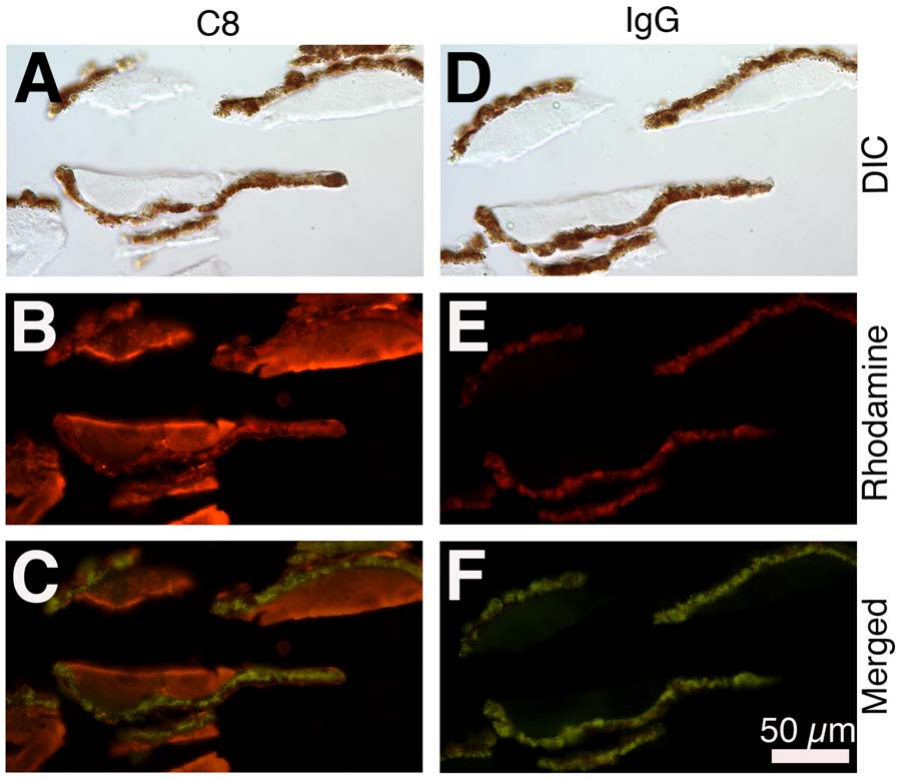
Complement factor 8 (C8) confirmed in drusen. Each column shows the same isolated RPE-capped drusen viewed with differential interference contrast microscopy (**A,D**), a rhodamine filter cube for immunofluorescence (**B,E**), and immunofluorescence merged with autofluorescence visualized with a fluorescein filter cube, resulting in a bronze -colored RPE (**C,F**). **A, B, C.** C8 immunoreactivity **D, E, F.** Control experiment using goat IgG.

To compare the overall protein content of RPE-capped drusen to their lipid content, total protein was determined using frozen (unfixed) drusen and a standard bicinchoninic acid assay, using albumin as a standard (**Table 4** **)**. Each RPE-capped druse contained 42.96±11.48 ng protein. A comprehensive examination of protein composition was achieved by mass spectrometric analysis of RPE-capped drusen and RPE. **Table 5** lists 20 proteins meeting quality criteria stated in the Methods. This list includes individual proteins that are well established as druse constituents (apoE, clusterin or apoJ, complement factor H, TIMP-3, and vitronectin). It also includes proteins associated with cellular function (ATP synthase β subunit, scavenger receptor B2, and retinol dehydrogenase 5) and previously described in RPE [41], [42], [43], [44]. Members of the final complement pathway comprising the membrane attack complex (components 5, 6, 8, 9) exhibited strong ion intensities, notably the 3 subunits of C8 (α, β, γ). C8 immunoreactivity was present in all drusen examined using immunofluorescence and an antibody recognizing the 3 subunits (**Figure 4**). Definitive identification of C8 within RPE was precluded by the intense autofluorescence and pigmentation of these cells.

**Table 4:** Protein Concentration and Mass in Drusen.

Case #	Drusen #	Total protein(ng)	Protein(ng/druse)	Protein(ng/µg druse)
15	482	15447	32.05	4.35
16	412	24482	59.42	1.95
17	262	10974	41.89	1.53
18	365	13725	37.60	1.98
19	276	15846	57.41	2.70
20	500	21030	42.06	2.20
21	410	12415	30.28	0.86
Mean	387	16274	42.96	2.22
SD	93	4836	11.48	1.10

**Table 5:** Proteins Detected in RPE-capped Drusen and RPE.

Protein Name	GeneSymbol	ID	Location*	%Coverage	# UniquePeptides
Albumin, serum	ALB	P02768	ES	6.7	6
Amyloid P component, serum	APCS	P02743	ES	31.8	8
Apolipoprotein E	APOE	P02649	ES	36.0	14
ATP synthase, H^+^ transporting, mitochondrial F1 complex, β polypeptide	ATP5B	P06576	Cyto	26.1	9
Clusterin	CLU	P10909	ES	9.0	1
Complement component 5	C5	P01031	ES	16.6	28
Complement component 6	C6	P13671	ES	15.4	11
Complement component 8, α polypeptide	C8A	P07357	ES	14.2	4
Complement component 8, β polypeptide	C8B	P07358	ES	11.8	3
Complement component 8, γ polypeptide	C8G	P07360	ES	47.8	6
Complement component 9	C9	P02748	ES	29.0	21
Complement factor H	CFH	Q03591	ES	17.7	3
Enolase 2 (γ, neuronal)	ENO2	P09104	Cyto	7.8	2
Forkhead-associated (FHA) phosphopeptide binding domain 1	FHAD1	B1AJZ9	Unk	0.0[^1^]	0
Major histocompatibility complex, class II, DR α	HLA-DRA	P01903	PM	27.6	5
Retinol dehydrogenase 5 (11-cis/9-cis)	RDH5	Q92781	Cyto	22.6	8
Scavenger receptor class B, member 2	SCARB2	Q14108	PM	2.5	2
Serum amyloid A1	SAA1	P02735	ES	0.0[^2^]	0
TIMP metallopeptidase inhibitor 3	TIMP3	P35625	ES	60.2	15
Vitronectin	VTN	P04004	ES	27.4	15

Notes: Locations assigned by Gene Ontology.

Abbreviations: ES: extracellular space; Cyto: cytoplasm; PM, plasma membrane; Unk, unknown.

* Location refers to location of most widely recognized function for each protein; even proteins in extracellular space begin within the endoplasmic reticulum of a source cell.

**Figure 5:**
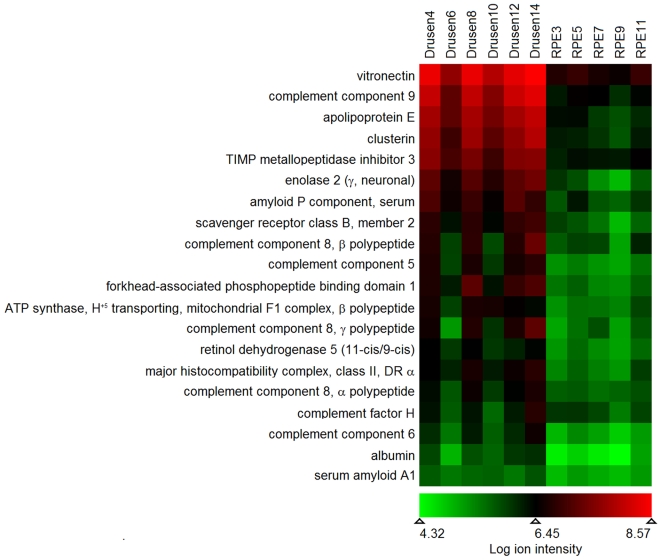
Proteins in RPE-capped Drusen and RPE. Spectral count ion intensities from mass spectrometry for each of 20 proteins meeting quality criteria (see Methods) from RPE-capped drusen (n = 6 eyes) and RPE (5 of the same 6 eyes). The ion intensity data was log transformed (base 10), visualized using the enhanced heat map feature in Mayday version 2.9 and sorted by druse signal strength. Similar proteins were detectable in drusen and RPE, with a log unit higher intensities in drusen.

To facilitate comparison of the relative abundance of 20 index proteins in RPE-capped drusen and RPE, mean ion intensities for all samples were plotted in a heat map, and sorted by druse signal strength (**Figure 5**). The proteins with the 6 highest ion intensities, which should not be confused with actual abundance, were vitronectin, complement component 9, apoE, clusterin (apoJ), TIMP-3, and serum amyloid A1. Overall, the protein profile was similar in RPE-capped drusen and RPE, with higher intensities and greater variability in the drusen, not surprising given the greater volume of the sample taken up by drusen. However, differences in intensities between RPE-capped drusen and RPE were on the order of a log unit, much greater than the 2-fold differences in volume described above. Thus, these proteins appear much more highly concentrated within drusen than in RPE.

Per druse, the combined weight of the lipids measured (112.42 ng, Table 2) was almost 3-fold larger than the weight of proteins (42.96, Table 3). Approximately equal quantities of EC, PC, and protein were detected. The average weight of an individual RPE-capped druse was 892 ng, so the total of 155.38 ng lipids and proteins accounts for 17.4% of total druse weight. The unaccounted-for weight includes both components not detectable by our methods and incomplete extraction of proteins and lipids.

## Discussion

Multiple levels of significance have been ascribed to the molecules trapped or sequestered in drusen. These include toxicity to the overlying RPE, stigmata of formative processes such as extrusion or secretion of cellular materials, extracellular enzymatic processing, cellular invasion or activity, and markers of a diffusely distributed disease process affecting RPE and BrM. We sought to identify abundant druse components under the assumption that such information would both permit research to focus on major contributory pathways and allow the informed construction of improved *in vivo* and *in vitro* model druse systems. Study strengths are the repeatability of results from large sample of eyes, the use of morphometric and analytic techniques, development of new protein extraction methods for archival preserved material, and the availability of previous studies against which our protein list could be validated (see below). Without more information about other druse components (e.g., carbohydrates or zinc [6] [12]), we cannot yet express abundance as proportions of total weight. Further, other assays will be required to identify and quantify the modified lipids and proteins [9], [45], [46] that are widely believed to accompany ARM progression.

We find that lipids, dominated by EC and PC, account together for 37–44% of druse content, on the basis of extraction with appropriate solvents and ultrastructural stereology. A much higher value for these components (2/3 of druse weight) determined through thin-layer chromatography and densitometry should be considered an upper limit and less definitive. It is possible that this larger proportion, obtained by normalizing results of protein and lipid assays by druse number, reflected an underestimate of total protein, operator-dependent differences in sample collection (see Methods), or limitations inherent in comparing assays of different precision. Further, our observation of high lipid enrichment of drusen relative to RPE is tempered by RPE PC content was low, considering PC comprises 20–40% of typical cellular phospholipids [47] Despite these uncertainties, we can safely conclude that EC and PC account together for at least 40% of druse content. Further, these 2 components are potentially accounted for by just one pathway, the retention of lipoproteins secreted by RPE [25] (see below). We also confirmed major druse proteins seen by others, suggesting the catalog of these components is nearing completion.

### Catalog of druse proteins

Protein identification in this study was challenged by the use of archival paraformaldehyde- preserved tissue and a sample (RPE–capped drusen) that was both small and of mixed origin. We developed novel methods that entailed de-crosslinking proteins. A key step in this procedure was a Tris-Glycine 1D PAGE gel to remove contaminants prior to mass spectrometry analysis. We sought to obviate concerns about mixed samples by also analyzing RPE isolated from the same eyes. We obtained robust signals for 20 proteins, 18 of which were common to RPE-capped drusen and RPE. Of direct interest to retinal lipid homeostasis was SCARB2. The class B scavenger receptors, SR-BI and SR-BII, are nearly identical, differing only at the C-terminus due to alternative gene splicing [48] [49]. Scavenger receptors, expressed by RPE [50], [51], bind plasma HDL, a postulated delivery route for carotenoid micronutrients essential for retinal and RPE health [52], [53]. Sequence variants in SCARB1 have been associated with increased risk for ARM [54].

In **Table 6**, we compare our 20 proteins to previous literature using proteomics or immunohistochemistry in native RPE and RPE-derived cell lines plus a recent description of the human RPE transcriptome [9], [13], [41], [43], [55], [56], [57], [58], [59], [60], [61], [62]. This comparison indicates that we detected many previously described druse proteins, and 18 of our 20 proteins match to the RPE transcriptome [62]. This comparison thus validates the new extraction and de-cross-linking techniques and solidifies the list of known major players by identifying the proteins that are abundant, survive fixation and cross-linking, and are readily ionizable. Collectively our data also support the idea that many druse proteins, although best characterized in other organ systems, are potentially produced locally within the eye. However, it is possible that RPE from donors without drusen, not examined herein, would not have as strong a signal for druse proteins. We emphasize that comprehensive proteomics in this study did not reveal some proteins revealed repeatedly by other methods, such as apolipoproteins B and A-I, amyloid (fibrillar and non-fibrillar), and complement component 3 [10], [11], [63], [64], thus underscoring the importance of replication via multiple approaches.

**Table 6:** Comparison with Other Studies.

GeneSymbol	DrusenProt	DrusenIHC	RPE Prot	LF Prot	Bleb Prot	mRNA
ALB	X		X	X	X	X
APCS		X				X
APOE	X	X				X
ATP5B	X		X	X	X	X
CLU	X	X	X			X
C5	X					X
C6	X					X
C8A	X					X
C8B	X					
C8G	X					X
C9	X		X			X
CFH		X				X
ENO2			X	X		X
FHAD1						
HLA-DRA		X				X
RDH5			X	X		X
SCARB2					X	X
SAA1	X					X
TIMP3	X	X		X		X
VTN	X	X	X	X		X

See Table 4 and Figure 5 for protein names.

Drusen Prot: proteomics [9]; Drusen IHC: immunohistochemistry [13], [60], [61]; RPE prot: native; human RPE proteomics [55]; LF prot: RPE lipofuscin proteomics [57]; Bleb prot: ARPE-19 cell lines exposed to hydroquinone [59]; mRNA: human RPE [62]; Individual proteins, by proteomics – ENO2, normal and diabetic human RPE [58]; ATP5B, human RPE mitochondria [41], [42] and human RPE melanolipofuscin [56] – and by immunohistochemistry – SCARB2 in monkey RPE [43].

Of the complement components in drusen, three subunits of C8 were detected by mass spectrometry and then confirmed using immunofluorescence. C8 belongs to the C5b-9 membrane attack complex, previously detected in drusen and BrM using antibodies recognizing the entire assembly [65], [66]. C8 is the only one of 31 complement components comprised of subunits (α, β, γ), all products of separate genes. A secreted disulfide-linked C8 α-γ dimer (which interacts with C5b-7) associates non-covalently with C8β (which then interacts with C9 monomers) [67]. Our data strengthen evidence for a role of this terminal complement component in druse pathogenesis.

### Mechanisms of lipid deposition in drusen and Bruch's

Here we found that EC and PC, two of the major lipid classes recently quantified for BrM lipoproteins [39], together accounted for at least 40% of druse volume. We could not obtain more details about the fatty acid distribution in these classes, as our samples were too small for comprehensive lipid profiling via liquid chromatography – gas chromatography using our previously published methods [39]. Thus, we could not directly compare fatty acids esterified to cholesterol in drusen to those in lipoproteins from plasma and BrM. Both of these lipoprotein sources have abundant cholesteryl linoleate (associated with dietary origin) and little cholesteryl docosahexanoate (associated with retinal origin) [39].

As revealed previously by filipin histochemistry, EC and UC are diffusely distributed through all drusen, with subregions of higher accumulation such as EC-rich shells, UC-rich cores, and lakes of pooled EC [19], [21], [68]. We previously showed electron dense particles in drusen post-fixed to preserve neutral lipid ([27], Fig. 6). Here we confirmed the lipophilic nature of these particles via extraction studies and showed that they are dispersed evenly throughout an apparently proteinaceous ground material. Both the size range and spatial distribution of the electron dense material are consistent with previous histochemical and ultrastructural findings and with the interpretation that these are lipoprotein particles like those in BrM, in either native or modified form. These data suggest a model of druse formation and enlargement involving trapping individual lipoprotein particles within a relatively more hydrophilic matrix, followed by particle fusion and lipid pooling.

While retention of RPE-secreted lipoproteins may account for much druse lipid, this process cannot account for all of it, because the composition we observed differs from lipoprotein composition by having both lower UC and higher fatty acids. Other sources must therefore contribute, and higher fatty acids may indicate post-mortem degradation of complex lipids (see [69]). Other explanations for this finding include a greater sensitivity of our chromatographic detection system for free fatty acids, or modification locally by enzymes such as lipoprotein lipase [70]. Proportionately less UC in drusen relative to lipoproteins was unexpected, in that evidence is accruing that proteins associated with RPE plasma and organelle membranes are shed into BrM and drusen [59], [71], [72], [73], [74], [75], [76], as long suspected [77]. Perhaps the UC content of those membranes is reduced before being shed.

Our focus on extra-macular drusen, dictated by convenience in isolating large quantities, does not preclude cautious inference from our conclusions to macular drusen, the universally agreed-upon predictors of ARM progression [5]. Elsewhere we demonstrated that macular drusen from ARM eyes contain abundant EC, UC, and apolipoproteins B and E [10], [21]. Soft drusen, dominant in the macula, contains a very high proportion of membranous coils. Originally termed membranous debris [78], this material has solid, neutral lipid cores when post-fixed for improved lipid preservation [22], prompting our suggestion of “lipoprotein-derived debris” as a better name. This term more accurately invokes a mechanism that could account for the presence of both UC and EC in these lesions [21], [22], [25], without prohibiting a role for other mechanisms for UC deposition (see above). Indirect evidence from the physical fragility of macular soft drusen suggests that these lesions may be proportionally more lipid-rich than the extra-macular drusen studied here [10], [29]. Thus, our estimate of extra-macular druse lipid content can be considered lower bounds for the content of the more fateful lesions in the ARM macula.

Within drusen, the association of lipid components plausibly derived from lipoproteins with strong signals from complement-related molecules is especially intriguing, given the multiple polymorphisms in complement factors that influence AMD susceptibility [14], [15], [16]. Our data suggest that lipoproteins in native or modified form, already described by others in human BrM [45], [46], [79], [80], [81], could be a potent stimulus for complement-mediated activation, as it is in atherosclerotic artery walls [82].

### Implications for model building and for treatment

Information from human eyes presented herein will help constrain and inform improved model systems for ARM. ARM-relevant choroidal neovascularization and cell loss have been productively studied in mouse models but *bona fide* drusen have been a more elusive target [83], [84]. Whether this situation is because mice lack maculas or because the relevant pathways for druse formation have not been identified remains uncertain. However, as EC and PC plausibly come to drusen as part of apoB lipoproteins secreted by RPE, just this one mechanism could account for 40% or more of druse volume. Manipulating genes encoding apolipoproteins pathways, including apoB, should therefore be a fruitful approach to faithfully replicating drusen with high EC content like those found in humans. Indeed, mouse models of age-related BrM lipid deposition based on such manipulations have been reported [85], [86], [87], [88], [89]. Conversely, our data also suggest that the contribution of any one protein to druse volume is likely small, and that in order to achieve abundant deposition, several druse proteins may have to be over-expressed, perhaps in combinations.

Our data also imply that direct mitigation of drusen via interference with biosynthesis and/or translocation of major druse components EC, UC, and PC should be contemplated as therapeutic routes in ARM. RPE re-populates BrM poorly following surgical extirpation of choroidal neovascular membranes in older patients [90], leading to intensive efforts to optimize the substrate for RPE survival by rejuvenating aged BrM [90], [91], [92]. In order for BrM to serve as a surgical bed for autologous grafts or stem cells to replace damaged RPE and photoreceptors [93], lipid-sequestering or -solubilizing approaches, using detergents, neutral pH cholesterol esterases, reconstituted HDL, apolipoprotein mimetics, and bioremediation should be included in BrM refurbishing [90], [94], [95], [96], [97].
